# Calculation of Mass Transfer and Cell-Specific Consumption Rates to Improve Cell Viability in Bioink Tissue Constructs

**DOI:** 10.3390/ma14164387

**Published:** 2021-08-05

**Authors:** Axel Pössl, David Hartzke, Peggy Schlupp, Frank E. Runkel

**Affiliations:** 1 Department of Life Science Engineering, Institute of Bioprocess Engineering and Pharmaceutical Technology, Technische Hochschule Mittelhessen—University of Applied Sciences, Wiesenstrasse 14, 35390 Giessen, Germany; axel.poessl@lse.thm.de (A.P.); david.hartzke@lse.thm.de (D.H.); 2 Department of Biology and Chemistry, Justus Liebig University, Ludwigstrasse 23, 35390 Giessen, Germany; 3 Department of Pharmaceutics and Biopharmaceutics, Philipps University, Robert-Koch-Strasse 4, 35037 Marburg, Germany

**Keywords:** 3D printing, extracellular matrix, diffusion, bioprinting, regenerative medicine, cell therapy, 1.1B4, cell culture, beta cell, biofabrication

## Abstract

Biofabrication methods such as extrusion-based bioprinting allow the manufacture of cell-laden structures for cell therapy, but it is important to provide a sufficient number of embedded cells for the replacement of lost functional tissues. To address this issue, we investigated mass transfer rates across a bioink hydrogel for the essential nutrients glucose and glutamine, their metabolites lactate and ammonia, the electron acceptor oxygen, and the model protein bovine serum albumin. Diffusion coefficients were calculated for these substances at two temperatures. We could confirm that diffusion depends on the molecular volume of the substances if the bioink has a high content of polymers. The analysis of pancreatic 1.1B4 β-cells revealed that the nitrogen source glutamine is a limiting nutrient for homeostasis during cultivation. Taking the consumption rates of 1.1B4 β-cells into account during cultivation, we were able to calculate the cell numbers that can be adequately supplied by the cell culture medium and nutrients in the blood using a model tissue construct. For blood-like conditions, a maximum of ~10^6^ cells·mL^−1^ was suitable for the cell-laden construct, as a function of the diffused substrate and cell consumption rate for a given geometry. We found that oxygen and glutamine were the limiting nutrients in our model.

## 1. Introduction

Tissue and organ defects are major challenges in the healthcare system, due to the shortage of donor material [1]. This can be addressed by tissue engineering, which is defined as the fabrication of biologically functional tissues in vitro using one of several biofabrication techniques [2]. One of the most investigated approaches is bioprinting, which can be executed by different techniques [3]. Cells are encapsulated in a hydrogel matrix (bioink) and printed into a three-dimensional (3D) structure. The bioink must provide an environment that ensures the sufficient supply of nutrients to cells for long periods in vivo, while the composition of the extracellular matrix (ECM) varies to fulfill specific tasks, depending on the tissue requirements. Summarized, the embedded cells interact with the ECM and other cells to promote attachment, migration, proliferation, or differentiation [4]. The ECM composition also affects the stiffness and the formation of concentration gradients and facilitates cell–cell interactions to form a functional tissue, as pointed out by Naba et al. [5]. Accordingly, the remodeling of the ECM can improve the viability and functionality of deteriorating tissues and organs.

Cell encapsulation in an artificial ECM, mimicking the conditions in vivo, requires the consideration of factors such as the graft site, cell type, and cell number required for therapy, as well as the ECM material composition, layer thickness, and their linked performance characteristics [6]. The selection of appropriate materials is essential for effective bioprinting and has been comprehensively discussed [7]. Despite these considerations, grafts often show a lack of long-term functionality or cell viability [8]. Major issues include the host immune response and protein adsorption on the material (biofouling), which creates an extra barrier for mass transfer. The continuous transfer of nutrients, metabolites, and cell products is inhibited, resulting in the undersupply of nutrients and the accumulation of waste products. Conditions that deviate from homeostasis cannot be tolerated for long, interfering with cell metabolism and ultimately causing cell death.

Assessment of the loading of bioink with cells is often evaluated by metabolic assays [9,10] or the differential staining of living and dead cells [9,11], following the bioprinting step. However, these experiments can only detect the potential for nutritional imbalance when the cells are already incorporated into the bioink hydrogel. The ideal number of cells for well-supplied cell-laden tissue constructs can be predicted before bioprinting using mass balance calculations for different geometries and spatial directions [12]. Process control management can then be used to establish pre-defined quality checkpoints before printing [13,14]. Accordingly, consumption and mass transfer studies for essential nutrients and metabolites should be carried out before printing, to increase the viability of the resulting bioink. Thus far, mass transfer studies have been published for simple hydrogels, with a single gelling agent [15], or a mixture of two materials; but more complex gelling agent mixtures, such as bioinks, have yet to be studied in detail. In contrast, the cell-specific consumption rates for standard cell lines have been reported by many researchers [16,17,18]. However, these should be verified under the experimental conditions unique to bioink applications, including differences in oxygen requirements for different cells and their organization [19].

To provide a theoretical framework for the generation of bioink constructs, we evaluated the diffusion coefficients for a complex hydrogel mixture and combined these with cell-specific consumption data to determine the cell number that can be supported in each construct. Our calculations were based on the equations for tissue constructs by McMurtrey [12], which we adapted for our application to determine the diffusion coefficients and cell consumption rates. We initially focused on the diffusion of the nutrients glucose and glutamine, their metabolites lactate and ammonia, and the electron acceptor oxygen. The model protein, bovine serum albumin (BSA), which mimics cellular products such as the hormone insulin, was used to characterize protein diffusion. Pancreatic 1.1B4 β-cell consumption rates were also determined for glucose, lactate, glutamine, and ammonia, allowing us to balance the nutrients mass transfer and consumption. Thus, we were able to calculate the ideal number of cells for an exemplary model implant in the treatment of type 1 diabetes under different conditions, by varying the concentrations of these molecules in two different media (normal cell culture media and blood).

## 2. Materials and Methods

### 2.1. Hydrogel Preparation

Hydrogels were prepared as previously described [20]. For diffusion experiments, the bioink (3.5% gelatin, 2.4% cellulose, 1.5% alginate, and 0.5% *(w/v*) carrageenan (all Sigma-Aldrich, Steinheim, Germany) was allowed to equilibrate at 37 °C for 1 h, followed by the application of 1 mL into rubber seals (Lux-tools, Wermelskirchen, Germany, inner Ø 25 mm, the specific height was determined experimentally) to fit the diffusion chamber. Accordingly, the rubber seals were placed on a plane area covered with Parafilm^®^ (Bemis Company, Neenah, WI, USA). The surplus hydrogel was removed with a microspatula, so that the height was uniform and corresponded to the height of the rubber seals. The bioink discs were cooled at 5 °C for 1 h. The discs were covered with 1 mL 100 mM CaCl_2_ (Carl Roth, Karlsruhe, Germany) overnight prior to the diffusion experiments.

### 2.2. Analytical Methods

Supernatants containing glucose and lactate were quantified using a Biosen C-line Analyzer (EKF Diagnostics, Barleben, Germany), according to the manufacturer’s protocol. Ammonia and glutamine titers were estimated with a K-GLNAM kit (Megazyme, Bray, Ireland), mostly according to manufacturer’s protocol, but extending the measuring time to 15 min for the second enzymatic reaction. The calibration ranges were set to 0.04–0.1 mg·mL^−1^ for ammonia and 0.1–0.7 mg·mL^−1^ for glutamine. The BSA concentration was determined using Roti-Nanoquant (Carl Roth, Karlsruhe, Germany) with a linear range of 2.5–100 µg·mL^−1^. For all kits, we used an HTX plate reader (BioTek, Friedrichshall, Germany) for absorption measurements. Dissolved oxygen levels were monitored with an OXY-4 mini meter (Presens, Regensburg, Germany) and OXY4 software (Version v2_11fb, 2011, Presens, Regensburg, Germany). For this purpose, we used an autoclavable flow-through cell setup (100 µL volume, Presens, Regensburg, Germany) equipped with an internal chemo-optical YAU-based sensor (Presens, Regensburg, Germany). The equipment was calibrated according to the manufacturer’s instructions at 25 °C.

### 2.3. Diffusion Experiments

**Figure 1:**
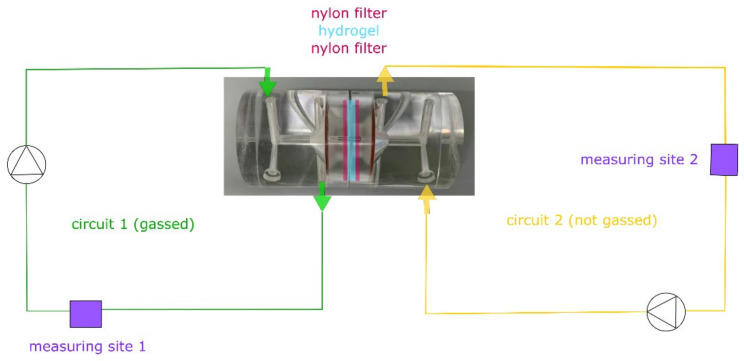
Schematic experimental setup for oxygen diffusion experiments using gassed and non-gassed circuits separated by a nylon filter (membrane) hydrogel sandwich and equipped with optical oxygen measuring sensors.

The diffusion of oxygen was measured using an Ussing chamber with two external circuits (Figure 1, Warner Instruments, Hamden, UK), whereas the other components were measured using a Franz diffusion cell (Gauer Glas, Püttlingen, Germany). The most suitable method for mounting the hydrogel between the donor and receptor chamber was to place the prepared bioink discs between two nylon filters (Ø 25 mm, 60 µm pore size; Merck, Darmstadt, Germany) and to tighten the apparatus with clamps. The integrity of the setup was confirmed using a sodium fluorescein solution (Thermo Fisher Scientific, Schwerte, Germany). The effective diffusion area (1.76 cm^2^) was the same for both setups. Before the experiments, the apparatus was allowed to equilibrate at the testing temperature for at least 30 min. To prevent drying, both setups were covered with Parafilm^®^ at relevant positions. The receptor medium was phosphate-buffered saline (PBS-1A; Capricorn Scientific, Ebsdorfergrund, Germany) plus 2.5 mM CaCl_2_.

Oxygen experiments (Figure 1) were carried out using 2.5 mg·mL^−1^ sodium sulfite (Thermo Fisher Scientific, Schwerte, Germany) as an additional compound in the receptor medium for the ungassed section. The donor and receptor volumes were 2 and 12 mL (Franz) or 20 and 20 mL (Ussing). Donor sites on the Franz diffusion cells were provided with the test substance dissolved in PBS-1A plus 2.5 mM CaCl_2_. For oxygen experiments, the circuit without sulfite was gassed with 95% (*v/v*) oxygen (Nippongas, Düsseldorf, Germany) at a rate of 4–6 L·h^-1^ using a Sho-Rate instrument (Brooks Instrument, Dresden, Germany) to form constant gas bubbles after equilibration, and flow-through cells for oxygen monitoring were added to each circuit. Homogenous mixing was ensured by level 3 peristaltic pumps (Masterflex, Gelsenkirchen, Germany).

For the other diffusion experiments (Franz), the receptor medium was mixed using a 9 mm magnetic bar (Carl Roth, Karlsruhe, Germany) at 200 rpm. Samples were taken at defined time points and replaced with fresh receptor medium. For each substance, a pre-experiment was carried out to estimate the approximate time period needed to achieve a linear mass transfer slope comprising at least five data points (r^2^ > 0.9), in order to work in a steady state region. The thickness of the bioink discs was ~1 mm (Ussing) or ~4 mm (Franz), and was determined by gravimetric measurement of the hydrogel after the experiment, assuming a cylindrical shape and the known density of the bioink [20]. Diffusion coefficients *D* were calculated as shown in Equation (1):

−*D* = Δ*m* × Δ*t*^−1^ × *A*^−1^ × *x* × Δ*c*^−1^ (1)

where Δ*m* × Δ*t*^−1^ is the mass of the diffused substance over time, *A* is the available area for mass transfer, Δ*c* is the concentration gradient, and *x* is the thickness of the bioink disc [21].

Diffusion coefficients of the substances in water were calculated as references, using the general Stokes-Einstein equation with Stokes hydrodynamic radii (glucose 0.365 nm [22], lactate 0.23 nm [22], ammonia 0.125 nm [23], glutamine 0.28 nm [24], albumin 3.51 nm [25], and oxygen 0.106 nm [26]) and common viscosities of water (25 °C, 0.891 × 10^−3^ kg·m^−1^·s^−1^; 32 °C, 0.758 × 10^−3^ kg·m^−1^·s^−1^; and 37 °C, 0.686 × 10^−3^ kg·m^−1^·s^−1^).

### 2.4. Cell-Specific Parameters

#### 2.4.1. Cultivation of 1.1B4 β-Cells

Pancreatic 1.1B4 β-cells (Sigma-Aldrich, Steinheim, Germany) were cultivated at 37 °C in a Heracell VIOS 160i incubator (Thermo Fisher Scientific, Schwerte, Germany) with an 8.5% CO_2_ atmosphere. The cells were cultivated in DMEM HXA high glucose (4.5 g·L^−1^) medium supplemented with 10% (*v/v*) fetal bovine serum and 4 mM l-glutamine (all from Capricorn Scientific, Ebsdorfergrund, Germany) to a confluence of ~80%. Subcultures were prepared with 0.05% trypsin/ethylenediaminetetraacetic acid (EDTA) solution (Capricorn Scientific, Ebsdorfergrund, Germany) for 5 min.

#### 2.4.2. Cell-Specific Growth and Production/Consumption Rates

We initially cultivated 15,000 cells per well in 24-well-plates (working volume =1 mL per well, surface area = 1.82 cm^2^) for up to 7 days, without media exchange. At defined times, cells were harvested from two wells to determine cell viability directly by cell detachment combined with trypan blue staining and the collection of supernatant samples, which were stored at −20 °C prior to testing. Cell-specific growth rates were calculated using Equation (2):

*µ*(*t*) = *Z*^−1^ × *dZ* × *dt*^−1^ (2)

where *µ* is the specific growth rate dependent on the time *t*, *Z* is the time-dependent absolute cell number, and *dZ* × *dt*^−1^ is the change in cell number over time. The exponential phase, and thus the maximum growth rate *µ*_max_, were estimated by graphical analysis.

During the exponential phase, we estimated the substance-specific production and consumption rates *q_x_* using Equation (3):

*q_x_* = −*Z*^−1^ × *dc_x_* × *dt*^−1^ (3)

where *dc_x_* × *dt*^−1^ is the substance-specific change in concentration over time.

### 2.5. Calculation of Cell Numbers in a Tissue Construct

Cell numbers were calculated for a 1D diffusion process (rectangular geometry, 10 cm × 1.0 mm × 1.0 mm) as previously described [12] for cell-laden tissue constructs, describing a model for unlimited diffusion into a tissue construct with a constant metabolic rate (zero-order metabolism). We modified this mass balance model equation by assuming stationary conditions for the diffusion process, as well as for the cell number, resulting in the elimination of time-dependent terms. For the summation series in sigma notation, we considered the two extreme values of ∞ and 1. As the effects were extremely small (lim → 0) for ∞ and +1 or –1 for the sinus term (*n* = 1), we eliminated the terms to derive the simplified model shown in Equation (4):

*Z* (cells·mL^−1^) = *D* × Δ*c* × *q*^−1^ × (0.5 × *x*^2^ − *T* × *x*)^−1^ (4)

where *x* is the linear depth into the construct, and *T* is the thickness of the construct. The estimated diffusion rate *D* and consumption rate *q* at 37 °C were utilized and varied so that two variables could be introduced for these terms, as shown in Equation (5):

*Z* (cells·mL^−1^) = (*α* × *D*) × Δ*c* × (*β* × *q*^−1^) × (0.5 × *x*^2^ − *T* × *x*)^−1^ (5)

**Figure 2:**
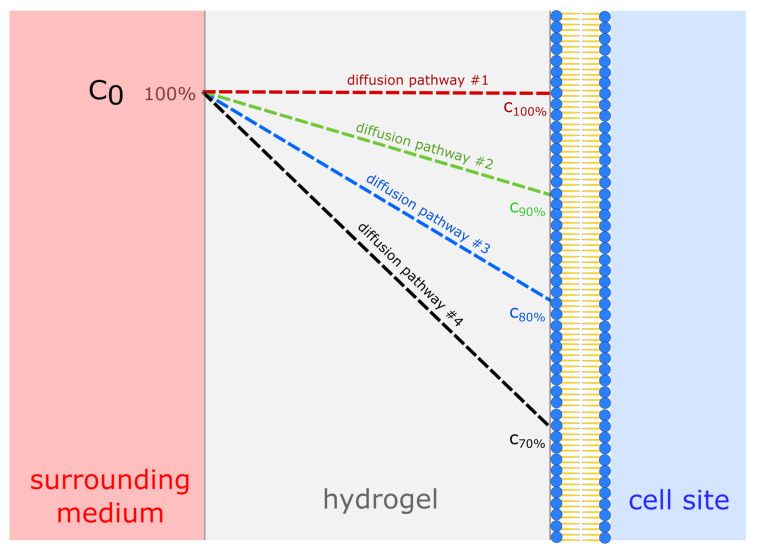
Visualization of potential substance distribution profiles dependent on the diffusion path length from the supply site to the cell site, varying from 100% to 70% of the surrounding medium. Thereby, *c_0_* represents the initial concentration of the surrounding medium.

Variable *α* is the amount of effectively diffused substance as a percentage of the initial value*,* which mimics the unexpected elongation of the diffusion pathway. Accordingly, *D* varied from 100% to 70% (Figure 2) due to local gradients or further inefficiency, in order to cover for deviations from 1D diffusion. Variable *β* is the minimum required nutrient status for the cells. Accordingly, *q* varied from 100% to 50% of the estimated value during exponential growth. The results of these calculations are presented as contour plots using Origin Pro software (Version v2019b, 2019, Originlab, Northampton, MA, USA), assuming all cells are located at a half thickness on average, reflecting their Gaussian distribution. We also assumed there would be no change in gel structure over time, or additional barriers. Metabolite removal was omitted because the production rates were assumed to be almost half the consumption rates and therefore not as important as the nutrient balance.

The initial concentrations in surrounding blood and cell culture medium are presented in Table 1. We used the oxygen unit calculation dv1_1 tool (Version 1.1, 2012, Presens, Regensburg, Germany) to convert the oxygen concentration values.

### 2.6. Data Analysis

All data were analyzed using OriginPro v2019b (Verison v2019b, 2019, Originlab, Northampton, MA, USA), with descriptive statistical tools. Mean values and standard deviations (SD) are presented, unless otherwise specified. Diffusion experiments with Franz cells were carried out with 10 diffusion cells (five cells on two days) at each temperature. Diffusion experiments with the Ussing chamber were performed four times on different days. Quantification assays were carried out at least in duplicate. Significance was tested using the non-parametric Kruskal–Wallis ANOVA at a level of *p* < 0.05 in OriginPro (Version v2019b, 2019, Originlab, Northampton, MA, USA).

## 3. Results

### 3.1. Diffusion Experiments

**Figure 3:**
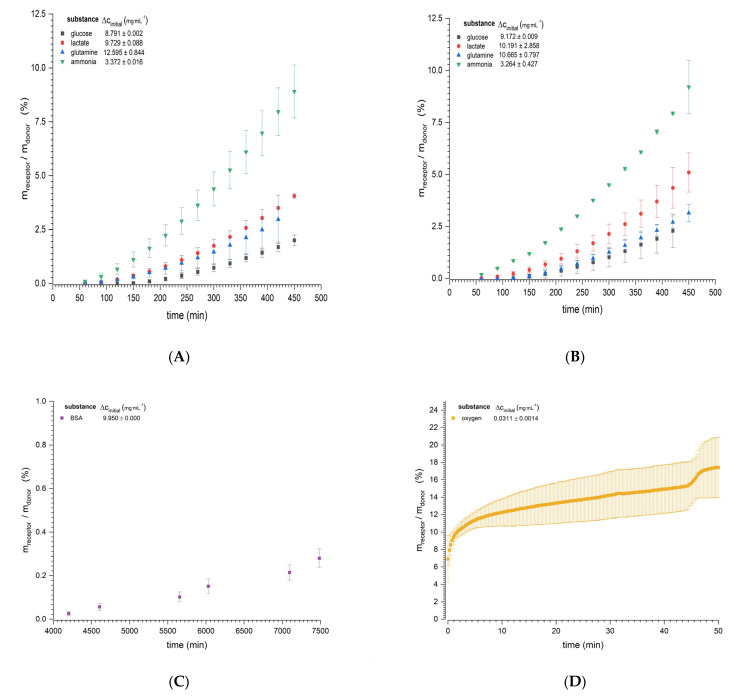
Mass transfer profiles for two different diffusion experiments as a quotient of the total mass in the receptor chamber and the initial mass in the donor chamber. The diffusion of glucose, glutamine, lactate, and ammonia through the nylon filter hydrogel sandwich is shown at (**A**) 37 °C and (**B**) 32 °C. (**C**) The diffusion of bovine serum albumin (BSA) is shown at 37 °C. All experiments in panels (**A**–**C)** were based on the use of 10 Franz diffusion cells. (**D**) Oxygen diffusion at 25 °C in an Ussing chamber. Initial concentration gradients are displayed in the upper left corner of each graph. Data are mean values ± SD (*n* = 10 for panels (**A**–**C**), *n* = 4 for panel (**D**).

The diffusion experiments showed continuously increasing mass transport through the bioink for all nutrients, metabolites, and the model protein BSA (Figure 3). The mass transfer reached a steady state following a system-dependent lag time. For diffusion experiments using the Franz cell (Figure 3A–C), a steady state was quickly reached by the smallest molecules (ammonia and lactate, starting at 150 min), but took longer for glucose (starting at 270 and 330 min at 37 and 32 °C, respectively). The longest time required to reach steady state was observed for the model protein BSA (approximately 70 h). The more quickly the steady state was reached, the higher the amount of permeated substance as a function of the concentration gradient. Ammonia was used at only one third of the concentration of the others, due to its toxicity for organs and tissues [31]. The smallest molecules (ammonia and lactate) were fastest, as evidenced by the steeper slopes and, thus, higher diffusion coefficients compared to glucose and glutamine, which permeated more slowly and where the total amounts were lower (Table 2). There was no evidence of temperature dependency based on the difference of 5 °C. BSA, as expected for the largest molecule, showed the lowest amount of permeation (0.28% of the initial amount at the donor site after 7500 min), but the increase was continuous. The diffusion of oxygen in the Ussing chamber (Figure 3D) had a lag time of 20 min, followed by a steady state for a further 20 min. The estimated diffusion rate for oxygen was 17.778 ± 7.32510^−10^ m^2^·s^−1^. During further progression of the experiment, a change in stability of the clamped hydrogel was observed, which was confirmed by the sudden increase of the oxygen passage after 45 min. The experiment was subsequently discontinued.

**Figure 4:**
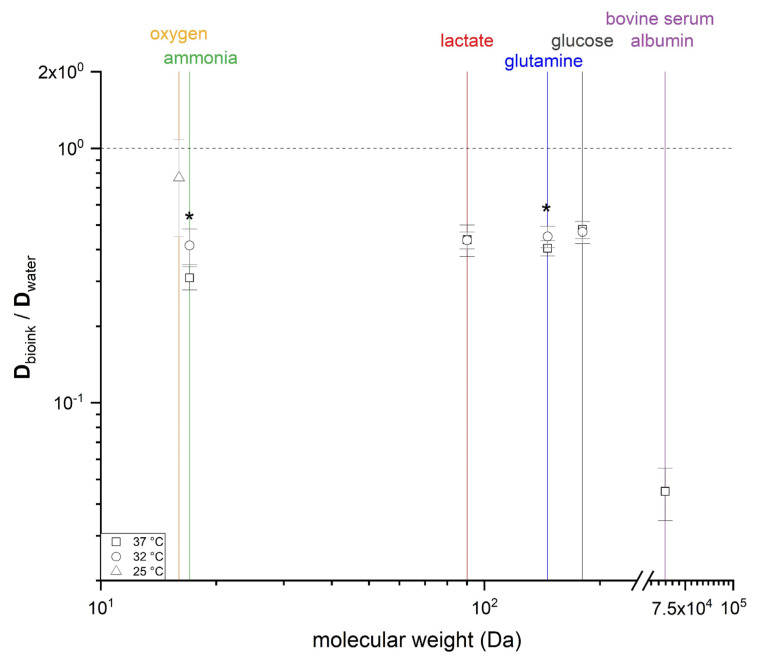
Effective diffusion coefficients of oxygen, ammonia, lactate, glutamine, glucose, and bovine serum albumin (BSA) through the bioink compared to water, as a function of the molecular weight and temperature. Data are mean values ± SD (*n* = 10 for all molecules except oxygen, where *n* = 4). Statistical significance was tested for the samples, differing in temperature, by Kruskal–Wallis ANOVA (* *p* < 0.05).

By comparing the diffusion coefficients of the substances through the bioink with the diffusion of the same substances in water, we were able to rank the efficiency of the diffusion process (Figure 4). The diffusion barrier of the hydrogel (bioink) was lowest for oxygen, despite the low experimental temperature. The diffusion of ammonia, a positively charged ion, was most affected by temperature, with diffusion efficiencies of 0.31 ± 0.03 (37 °C) and 0.42 ± 0.07 (32 °C), followed by glutamine 0.45 ± 0.04 (37 °C) and 0.41 ± 0.03 (32 °C), each statistically significant. Lactate and glucose showed no significant temperature dependency, and the diffusion efficiencies ranged from 0.44 to 0.48, representing a difference in diffusion rate of 52–56% compared to water. The high molecular weight of BSA (65 kDa) reduced the efficiency of transport through the bioink material by 95%.

### 3.2. Cell-Specific Parameters

**Figure 5:**
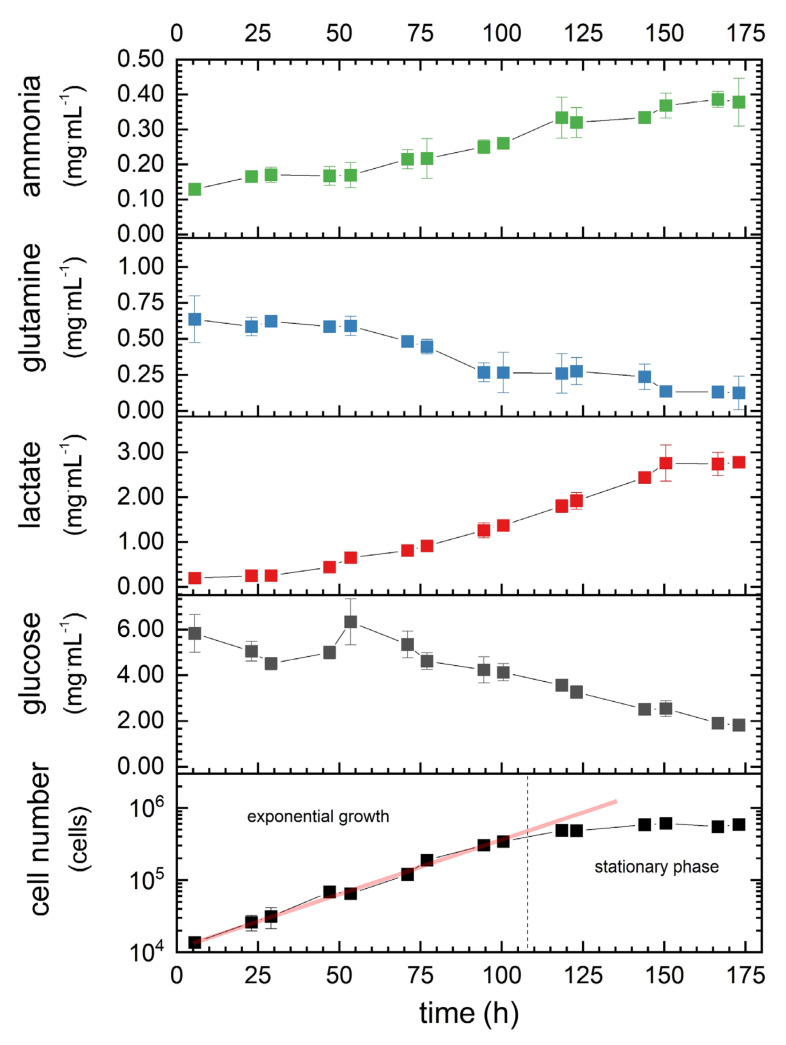
Growth kinetics of 1.1B4 β-cells (bottom chart), with corresponding concentrations of glucose and glutamine and their metabolites. Data are mean values ± SD (*n* = 3).

During cultivation without media exchange, the 1.1B4 β-cell number increased continuously up to 100.5 h, representing the exponential growth phase (Figure 5, bottom chart). The maximum cell number (610,000 cells·mL^−1^) was observed after 173 h, with a maximum cell-specific growth rate of 0.04 h^-1^ (Table 3) and a doubling time of 19 h. The nutrients (glucose and glutamine) were continuously consumed by the cells, while the corresponding metabolites (lactate and ammonia) accumulated. At the end of the experiment, glutamine was nearly depleted (80.5% consumed), whereas glucose levels were reduced to 68.7% of the initial amount. The rate of glucose consumption during the exponential growth phase was 6.3-fold higher than that of glutamine (Table 3). The lactate production rate during the same phase was 40% lower than the glucose consumption rate, whereas the ammonia production rate was 54% lower than the glutamine production rate. Remarkably, lactate reached a plateau at a concentration of 2.761–2.784 mg·mL^−1^ after 150.5 h (during the stationary phase). The consumption rates (*q*) and diffusion coefficients (*D*) were then used and varied to calculate cell numbers that could be supplied efficiently in a model tissue construct for different conditions.

### 3.3. Calculations of Cell Numbers in a Tissue Construct

**Figure 6:**
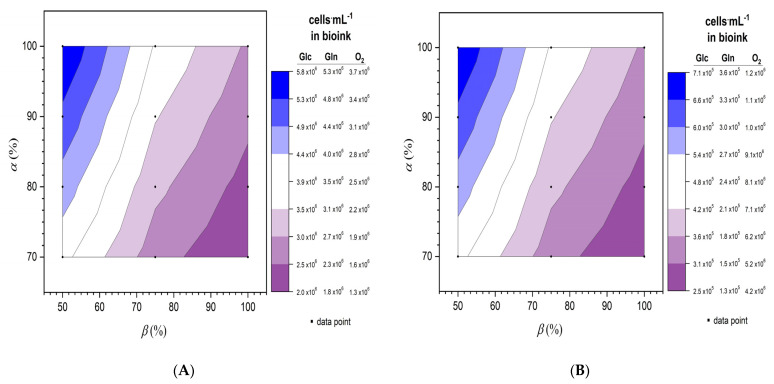
Calculation of cell numbers that can be supported in a model tissue construct of defined geometry (**A**) in cell culture medium and (**B**) in blood for the three nutrients glucose (Glc), glutamine (Gln), and oxygen (O_2_), based on variations in diffusion efficiency (*α*) and consumption rates (*β*).

Given the empirical values for mass transfer and consumption rates determined above, we calculated the cell numbers that could be adequately supplied in a model tissue construct. Two conditions were evaluated, differing in the initial values of the surrounding medium (Figure 6). The calculation was carried out for different percentages of *D*(*α*) and *q* (*β*). The maximum number of adequately supplied cells in a construct surrounded by cell culture medium (Figure 6A) was 5.8 × 10^6^ cells·mL^−1^ based on the data for glucose, and the minimum number was 1.3 × 10^6^ cells·mL^−1^ based on the data for oxygen, indicating that oxygen supply is the main limitation. Oxygen thus limits the number of cells in the tissue construct to a value between 1.3 × 10^6^ and 3.7 × 10^6^ cells·mL^−1^ if the cells are surrounded by culture medium. In contrast, with blood as the surrounding medium (Figure 6B), the maximum number of adequately supplied cells was 1.2 × 10^6^ cells·mL^−1^ based on the data for oxygen, and the minimum number was 1.3 × 10^5^ cells·mL^−1^ based on the data for glutamine, indicating that glutamine is the limiting factor. Glutamine thus limits the number of cells in the tissue construct to a value between 1.3 × 10^5^ and 3.6 × 10^5^ cells·mL^−1^ if the cells are surrounded by blood.

## 4. Discussion

To maintain cell viability, the preparation of cell-laden constructs must take into account the properties of the hydrogel matrix (e.g., the physicochemical properties of the gelling agents), as well as cell-specific characteristics such as nutrient consumption rates. Cell compatibility and encapsulation are often addressed experimentally by monitoring cell viability during long-term cultivation once the construct has already been formed [9,10,11]. However, if the initial cell number (and by extension, the nutrient consumption rate) is not compatible with mass transfer limitations, then the cells will experience stress, and may even die [33]. Cell-laden constructs with a large diameter have been shown to trigger necrosis, which induces an immune response that can lead to construct rejection [6]. Oxygen limitation is a key restriction affecting cell numbers, and balancing the mass transfer and consumption of nutrients (and their metabolites) is therefore necessary, to avoid conditions that inhibit cell growth [16].

The additional diffusion barrier caused by cell encapsulation depends on the nature and concentration of the polymer(s) in the matrix [34]. Experiments with nutrient-spiked hydrogels provide a simple way of estimating the differences between diffusion coefficients in the hydrogel and water [35]. However, the driving force of a concentration gradient for diffusion is dependent on the initial concentration in the surrounding medium and the penetration time. Accordingly, mass transfer will not remain in a steady state for long due to the small volume of the receptor medium (the hydrogel). In addition, we performed steady state mass transfer studies with different nutrients at different temperatures to imitate a range of graft sites. We estimated diffusion coefficients for glucose and other small molecules (in terms of hydrodynamic volume, 0.106–0.361 nm Stokes radius) in the context of a hydrogel with a total gelling agent concentration of 7.9% (*w/v*). The glucose diffusion coefficients we obtained were 4.3564 × 10^−10^ at 37 °C and 3.7937 × 10^−10^ m^2^·s^−1^ at 32 °C, indicating the gel we used was slightly less permeable compared to previous studies using 2% *(w/v*) alginate gels (6.4 × 10^−10^ m^2^·s^−1^ at 30 °C) [15], due to the higher polymer content. Similarly, our findings for larger molecules such as BSA (3.61 nm Stokes radius) were also lower compared with published reports [36].

We summarized the barrier function of the bioink by evaluating the efficiency of diffusion compared to water. The plot showed a primary dependency on molecular weight (and thus hydrodynamic volume), but charged molecules appeared to interact more with the bioink matrix due to the physicochemical properties of the polymers. Differences in temperature appeared to have a less significant effect. Our data agree with previous studies reporting a lower diffusion rate in more concentrated gels [34]. The permeation efficiency may also be affected by the average pore size, which can be estimated by rheology [37] or thermoporometry, using differential scanning methods [38]. The average pore size of our gel was ~8.94 nm, based on our previous report [20] and the equations proposed by Devi et al. [37]. This may explain the prolonged diffusion time (and thus smaller diffusion coefficients) for BSA, reflecting the similar size of the BSA molecule and the average pore. Future studies should also consider additional barriers to encapsulation [39], such as biofouling [40] or laminar boundaries, to ensure that implant sites resemble in vivo conditions as closely as possible.

Having characterized the mass transfer properties of the hydrogel, we measured the growth, consumption, and production rates of the pancreatic β-cell line 1.1B4 (Figure 5). After an exponential growth phase of the cells, a stationary phase occurred. This phase was characterized by a steady state of cell proliferation and cell death, due to the limited supply, production, and accumulation of products or metabolites and the available growth area for expansion of the cells. Our empirically determined growth rate of 0.04 h^−1^ during the exponential phase agreed with previous findings for the same cells (0.035 h^−1^) [19]. The values were slightly higher than reported for other standard cell lines, such as hybridomas (0.03 h^−1^) [16], Chinese hamster ovary cells (0.023 h^−1^) [18], and baby hamster kidney cells (0.021 h^−1^) [17]. The glucose consumption and lactate production rates were similar to those previously reported for murine hybridomas: 1.35·10^−11^ mg·s^−1^·cell^−1^ glucose and 1.13·10^-11^ mg·s^−1^·cell^−1^ lactate [16]. Previous experiments with 1.1B4 pancreatic β-cells consumed a 6.75-fold lower value for glucose [19], which may reflect the consumption rate dependency of concentration [16]. Our glutamine consumption and ammonia production rates were in the mid-range compared to values previously reported for standard cell lines and may also depend on the initial concentration [16]. Generally, the initial concentration of the carbon and nitrogen sources and their metabolites strongly influences the consumption and production rates [16,17,18,41], and it is therefore possible to draw only approximate comparisons between the different cell lines.

The mass transfer and cell consumption data can be used to estimate the ideal cell number in a model tissue construct to ensure an adequate supply of nutrients. Based on initial concentrations in blood and cell culture medium, we found that glutamine is the limiting substrate in vivo (blood), whereas the differences among substrates in vitro (cell culture medium) are less pronounced (Figure 6). In the cell culture medium, the number of cells per milliliter ranged from 2.9 × 10^6^ (glucose) to 1.8 × 10^6^ (oxygen), assuming no further influences on diffusion (100% *α*) or consumption rates (100% *β*). However, excess substrates in the cell culture medium have limited relevance to the situation in vivo, and this must be addressed in the context of implants and determined for each individual approach, as each cell line behaves differently, and viability may differ before and after the biofabrication process [42]. Moreover, the assumed static cell culture differs from the in vivo environment, which features a systemic circulation and a more complex linked network of biochemical pathways. For example, glutamine supplies may increase in vivo due to the balance between glutamine and glutamate [28], which would make oxygen the limiting factor [19].

Our calculations provide the designer of tailored artificial ECMs with insight into the number of cells that can be encapsulated in a model construct, without complex interactions or geometries. The limiting mass transfer of diffusion is separated from the usually faster mass convection to minimize terms and focus on the slowest processes. In this context, the efficiency of the diffusion kinetics across the hydrogel and the efficiency of the microkinetics (diffusion of the substrate from the cell membrane to the intracellular site of substrate conversion) can be varied to evaluate their impact, based on known data. Our theoretical approach also allows for more complex interactions, such as additional diffusion barriers or connections between substances, as shown for the dependency of cell growth on the concentration of glucose and glutamine [43]. Transient diffusion and cell consumption conditions can also be addressed by adapting the general equations [12] and modifying them according to user assumptions. In addition, it should be noted that the metabolic behavior of the cells can be altered by the surrounding environment [12], in this particular case the bioink. Furthermore, gel aging during cultivation [20], which can also influence diffusion, could be included to model the behavior of the gel over longer periods.

Based on our data, we can evaluate the feasibility of a pancreas implant containing 1.1B4 β-cells. The average human pancreas weighs 90.31 g (male) or 84.88 g (female) [44]. Assuming the mean overall weight (87.6 g) and combining the average volume of a human pancreas (45 cm^3^) [45] with the relative abundance of islets of Langerhans (4.487% of the pancreas) [45], the cell volume is 2.02 cm^3^ with a mass of 3.93 g. A β-cell fraction of 57.13% for one islet of Langerhans [45] leads to a required total mass of 2.25 g of cells. The mass of a HeLa cell is 2–3 ng [46], indicating that ~10^9^ cells would be required. Based on the calculated values for blood supply (Figure 6, 100% for *α* and *β*), the number of 1.1B4 β-cells that can be supported ranges from 1.8 × 10^5^ (glutamine) to 6.0 × 10^5^ (oxygen) cells per milliliter, equating to between 17,800 and 30,000 cells in total for the model construct. Thus, complete replacement of β-cells would therefore require between 33,333-fold and 56,180-fold the length of the model construct (3333 and 5620 m). Using a tissue model with ten 1 mm layers would reduce this length to 333 and 562 m, with required areas of 0.333 and 0.562 m^2^, respectively. Implantation sites for islets of Langerhans include the kidney, liver, muscle, and omentum [47]. The latter is the preferred site because it provides an area of 0.0825 m^2^ (25 cm × 33 cm) [48]. This area is still 75–85% below the requirement, but the deficit might be addressed by combining with other implantation sites such as muscles to restore the full quantity of β-cells in normoglycemic patients. Other approaches include the use of devices such as a beta-air device to supply cells [49], the modulation of consumption by reducing nutrient or metabolite concentrations, or the induction of neovascularization [50].

## 5. Conclusions

In conclusion, we were able to balance a determined mass transfer through a bioink for three essential nutrients and cell consumption rates by simplified equations, based on general models for calculating well-supplied cell densities in model bioprinted constructs under variation of cultivation, diffusion, and cell consumption conditions. The goal of identifying the limiting substrate was achieved, and thus a tissue construct can be easily optimized before bioprinting has even occurred. Our work provides a framework for other bioprinting researchers to predict substrate limitations and cell densities for bioprinting, reducing the need for frequent post-printing cell viability assessments from a quality assurance perspective.

## Figures and Tables

**Table 1:** Summary of initial concentrations of essential nutrients for cells in blood and cell culture medium, with references.

Substance	Blood Values	Cell Culture Medium
Glucose	~1170 mg·L^−1^ [27]	4500 mg·L^−1^
Glutamine	~3.73 µg·mL^−1^ [28]	27.4 µg·mL^−1^
Oxygen	~4.5 mg·L^−1^ [29]	6.95 mg·L^−1^ (36 °C) [30]

**Table 2:** Diffusion coefficients calculated for essential nutrients, their metabolites, and the model protein bovine serum albumin (BSA) migrating through bioink (data from Figure 3). Data are mean values ± SD at two different temperatures. Each experiment involved *n* = 10 Franz diffusion cells, or *n* = 4 repeats in an Ussing chamber in the case of oxygen diffusion.

Substance	Temperature (°C)	*D*_experimental ×_ 10^−10^ (m^2^·s^−1^)
Glucose	37	4.3564 ± 0.3407
	32	3.7937 ± 0.3807
Lactate	37	6.3021 ± 0.8930
	32	5.5772 ± 0.4195
Ammonia	37	8.1957 ± 0.8517
	32	9.7921 ± 1.5702
Glutamine	37	4.7901 ± 0.3353
	32	4.7368 ± 0.4589
BSA	37	0.0423 ± 0.0098
Oxygen	25	17.778 ± 7.325

**Table 3:** Summary of cell-specific growth data based on the consumption and production rates of 1.1B4 β-cells during the exponential growth phase. The value for cell-specific oxygen consumption is an average reported for human promyelocytic leukemia (HL60) cells [32].

Parameter	Value
*µ*_max_	0.04 h^−1^
*t*_double_	19 h
*q*_glucose_	−2.6987 × 10^−11^ mg·s^−1^·cell^−1^
*q*_lactate_	1.6082 × 10^−11^ mg·s^−1^·cell^−1^
*q*_glutamine_	−0.4261 × 10^−11^ mg·s^−1^·cell^−1^
*q*_ammonia_	0.1943 × 10^−11^ mg·s^−1^·cell^−1^
*q*_oxygen_ [32]	−0.0267 × 10^−11^ mg·s^−1^·cell^−1^

## Data Availability

All the data is available within the manuscript.
